# Detection of *Theileria orientalis* genotypes in *Haemaphysalis longicornis* ticks from southern Australia

**DOI:** 10.1186/s13071-015-0839-9

**Published:** 2015-04-16

**Authors:** Jade Frederick Hammer, David Emery, Daniel Ross Bogema, Cheryl Jenkins

**Affiliations:** Faculty of Veterinary Science, University of Sydney, Sydney, NSW AUS; The ithree institute, University of Technology Sydney, Broadway, Sydney, NSW AUS; Elizabeth Macarthur Agricultural Institute, New South Wales Department of Primary Industries, Menangle, NSW AUS

**Keywords:** *Haemaphysalis longicornis*, *Theileria orientalis*, Ikeda, Buffeli, Chitose, theileriosis

## Abstract

**Background:**

*Theileria* are blood-borne intracellular protozoal parasites belonging to the phylum Apicomplexa*.* Previously considered a benign parasite in Australia, outbreaks of clinical disease resulting from *Theileria orientalis* genotypes have been reported in Australia since 2006. Since this time, outbreaks have become widespread in south-eastern Australia, resulting in significant adverse impacts on local dairy and beef industries. This paper provides the first investigation into the possible biological and mechanical vectors involved in the rapid spread of the parasite.

**Methods:**

To identify possible vectors for disease, ticks, biting flies and mosquitoes were collected within active outbreak regions of Gippsland, Victoria. Ticks were collected from cattle and wildlife, and mosquitoes and biting flies were collected in traps in close proximity to outbreak herds. Ticks were identified via DNA barcoding of the mitochondrial cytochrome oxidase I gene. Barcoded ticks were pooled according to species or phylogenetic group and tested for the presence of *T. orientalis* and the genotypes Ikeda, Chitose and Buffeli using real-time PCR.

**Results:**

DNA barcoding and phylogenetic analysis identified ticks from the following species: *Haemaphysalis longicornis*, *Ixodes holocyclus*, *Ixodes cornuatus*, *Ixodes hirsti*, and *Bothriocroton concolor*. Additional *Haemaphysalis*, *Ixodes* and *Bothriocroton* spp. were also identified. Of the ticks investigated, only *H. longicornis* ticks from cattle carried theilerial DNA, with the genotypes Ikeda, Chitose and Buffeli represented. Mosquitoes collected in close proximity to outbreak herds included; *Aedes camptorhynchus, Aedes notoscriptus, Coquillettidia linealis, Culex australicus,* and *Culex molestus.* Low levels of *T. orientalis* Buffeli genotype were detected in some mosquitoes. The haematophagous flies tested negative.

**Conclusions:**

This is the first demonstration of a potential vector for *T. orientalis* in the current Australasian disease outbreak.

## Background

*Theileria* are blood-borne intracellular protozoal parasites belonging to the phylum Apicomplexa. Historically, the precise classification of the various genotypes of the parasite has been the cause of some confusion [1-3]. In particular, the agent of the more benign form of bovine theileriosis was speciated differently, being named as *T. buffeli* in Australia, *T. sergenti* in Japan and East Asia, and *T. orientalis* in many other locations [2]. The name Theileria orientalis is now widely applied to the species present in Australia, New Zealand and throughout Asia [4-15]. It is now appreciated that *T. orientalis* can be separated into several genotypes, namely, type 1 (Chitose), type 2 (Ikeda), type 3 (Buffeli), types 4–8 [6,7] and types N1-N3 [14].

*Theileria* has been recognised in Australia since 1910 [16] and is found in all states except Tasmania [8]. *T. orientalis* has long been considered a benign parasite [4,6,16,17]. However, pathogenic genotypes are now recognised in many countries including Australia [7,10], New Zealand [5], Japan, [11], China [18] and Korea [19]. Since 2006, a large number of outbreaks of clinical disease have been reported in New South Wales, Victoria, Western Australia [7,10,20], and more recently South Australia [21]. The emergence of clinical theileriosis and associated mortalities in Australia is of increasing concern for the local beef and dairy industries [7,8]. Clinical signs of *T. orientalis* are mostly associated with the sequelae from anaemia. These signs can include lethargy, weakness, anorexia, pale mucous membranes, lymph node swelling, tachypnoea, tachycardia, dyspnoea, jaundice, late-term abortion, dystocia, pyrexia, and mortality [5,7,8].

The method of introduction of *Theileria* to Australia is uncertain and may have occurred with the introduction of *Haemaphysalis longicornis* ticks into Australia [16,22,23]. Moreover, the emergence of more pathogenic genotypes remains unclear, although it has been speculated that the Ikeda genotype was introduced to Australia from cattle imported from Eastern Asia [24]. A severe outbreak of theileriosis on a beef farm has been linked to *T. orientalis* Ikeda and Chitose genotype entering the more southern, temperate area of Victoria [7]. It is known that the Ikeda genotype is more associated with clinical disease than Chitose and Buffeli in Australia, and this also occurs internationally where clinical cases of *T. orientalis* have been reported [10,11]. Currently, the Australian vectors for *T. orientalis* Ikeda genotype have not been determined.

*Haemaphysalis longicornis,* a known vector tick for *T. orientalis* in other countries [2], has a complex life cycle. It has a wide host range, and a distribution along the coastal strip from south east Queensland to the Victoria border with rare occurrences in other locations in Victoria [25]. It has also been reported in locations in south west Western Australia [26]. It is a three host tick with larva, nymph, and adult engorging for approximately 7 days before dropping from the host [27]. Female ticks lay up to 2000 eggs in late spring and early summer (November to January) which hatch in 60–90 days depending on environmental conditions. All stages can survive more than 200 days without feeding [27]. Most adults are seen in January and February, reproduction is via parthenogenesis, and so male adults are rarely seen. Other ticks recorded from cattle in Victoria include; *Ixodes cornuatus, Ixodes holocyclus, Amblyomma moreliae, Bothriocroton concolor* (formerly *Aponomma concolor*)*, Aponomma fimbriatum, Bothriocroton hydrosauri* (formerly *Aponomma hydrosauri*)*,* and *Rhipicephalus sanguineus* [25,28]. Of these ticks, *I. cornuatus* and *I. holocyclus* are known from Eastern Victoria where this research was conducted [29]. In addition, *Amblyomma moreliae* has also been recorded in Gippsland [25]. *Bothriocroton concolor* is mostly seen on echidnas, *Bothriocroton hydrosauri* is mostly found on reptiles, and *Rhipicephalus sanguineus* is mostly seen on dogs and the occurrence of all of these ticks on cattle is rare [25]. The role of *H. longicornis* has been investigated as a vector of “*T. buffeli”* in Australia. This species was once implicated as a likely vector [30], but was discounted in subsequent studies [1,22]. Attempts to experimentally transmit *T. orientalis* Warwick strain (Buffeli type) to cattle via the trans-stadial and transovarial routes were unsuccessful while trans-stadial transmission was routinely achieved with *H. humerosa* [22,31]. Conversely in Japan, experimental transmission studies employing the Japanese Ikeda genotype of *T. orientalis* with *H. longicornis* sourced from both Australian and Japanese populations were successful. Indeed, these early studies were used as one criterion to differentiate the Australian “*T. buffeli*” (now referred to as *T. orientalis* Buffeli) from “*T. sergenti*” which encompasses the Ikeda genotype [2]. In separate experiments, the native Australian wallaby tick, *H. bancrofti*, was shown to transmit both *T. orientalis* Ikeda [2], and a strain of *T. orientalis* sourced from Queensland and presumed to be the Buffeli type “Warwick strain” [32]. Both *H. bancrofti* and *H. humerosa* are apparently competent vectors for *T. orientalis* under experimental conditions, but the range of both of these species is principally limited to the more temperate and tropical climates of the northern coast of New South Wales and coastal Queensland. Although *H. bancrofti* has been recorded sporadically from Victoria [33] this tick and *H. humerosa* are less likely to act as vectors in Victoria. Taken together, these data suggest that the most likely vectors of *T. orientalis* might include *H. longicornis, H. bancrofti, I. cornuatus, I. holocyclus,* and *A. moreliae.*

## Methods

### Sample collection

**Figure 1:**
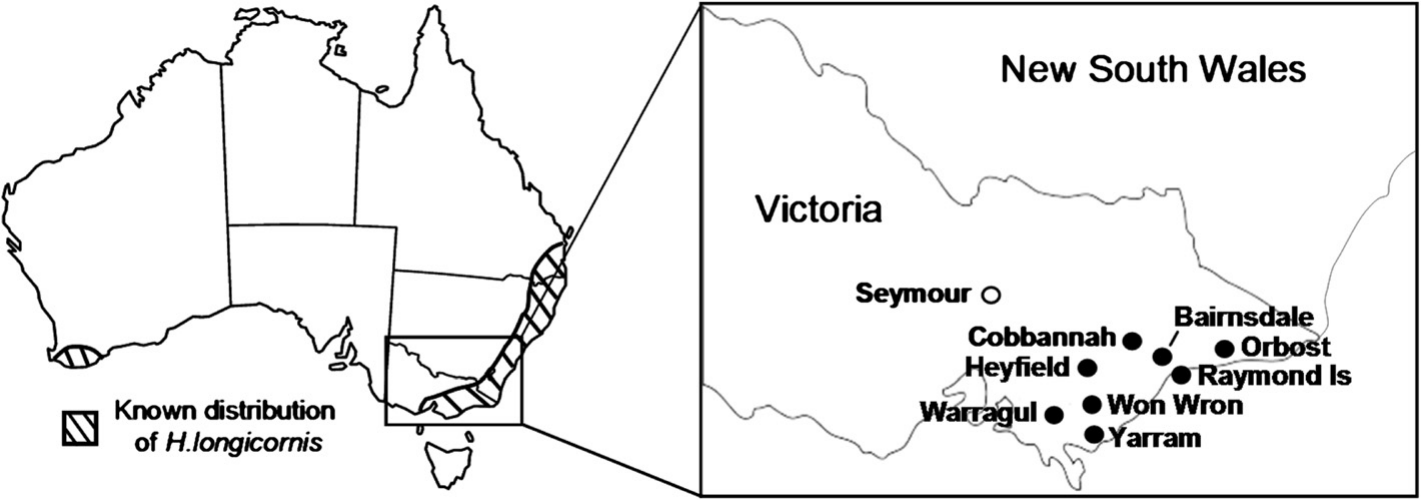
Distribution of *Haemaphysalis longicornis* in Australia. Map of Australia showing the known range of *H. longicornis* as described in [30]. An enlarged map of the state of Victoria is also shown with the geographic locations, from which ticks and other arthropods were collected, highlighted (closed circles). The location of the likely entry point of *T. orientalis* Ikeda into the state of Victoria is also shown (open circle).

The collection of ticks and arthropods invertebrates described did not require ethics approval. Local producers and wildlife experts provided ticks taken from animals in their care. For this study, 220 ticks were collected from a variety of livestock and wildlife hosts over a large geographical region of Victoria (Figure 1) by local veterinary practices, wildlife carers, farmers and members of the public. Ticks were predominantly of the adult stage, although some larval and nymph stage ticks were also collected, and ranged from unengorged through to fully engorged. Haematophagous flies were collected during suitable weather during summer 2013, autumn 2014, and summer of 2015, in locations within one kilometre of outbreak farms. Two Nzi traps (Rincon-Vitova Insectaries, Ventura, CA, USA) were set up using dry ice as an attractant with 1-Octen-3-ol. A total of 4 (UV) light traps (Australian Entomology Supplies, Model PR101) were used for the collection of mosquitoes. Each trap was baited with carbon dioxide from dry ice. Traps were set on properties where clinical cases of theileriosis had been identified, or located in close proximity not more than 1 kilometre from such a herd. Capture data was recorded for all arthropods, which were then frozen and stored at −80°C until required.

### Arthropod identification

Mosquitoes were identified morphologically using a dichotomous key [34]. Mosquito species identified included *Aedes camptorhynchus, Aedes notoscriptus, Coquillettidia linealis, Culex australicus,* and *Culex molestus.* Preliminary identification of ticks was undertaken using identification charts [25]. All ticks collected were subsequently transported on dry ice to the laboratory for molecular identification. Ticks were photographed, in order to maintain a record of sample morphology, and samples for which morphological identification was uncertain (n = 135), were subjected to DNA barcoding. A *H. longicornis* tick from an Australian reference collection (AgriBio, Centre for AgriBioscience) was also barcoded for comparison with the field-collected ticks. For PCR testing, an individual leg was dissected from each tick using separate sterile forceps and scalpel blades and the legs were washed with PBS to remove any adherent host material. DNA was extracted from the tick legs using the DNeasy Blood and Tissue kit (Qiagen) according to the manufacturer’s instructions and with a 100 μL elution volume. A process control which contained no tissue was included with each DNA extraction run to control for cross-contamination of samples. The mitochondrial cytochrome oxidase I (COI) genes were amplified using the metazoan “barcoding” primers of Folmer [35]. PCR amplification was performed in a total volume of 25 μL comprising 1 × Immolase reaction buffer, 2.0 mM MgCl_2_, 200 μM dNTPs, 400 nM each of the LCO1490 and HCO2198 primers and 1 U of Immolase DNA polymerase (Bioline). PCR products were visualised on a 0.5 × TBE-1.5% agarose gel and of the 135 ticks tested, 105 yielded sufficient amplicon for downstream sequencing. PCR products were purified using the Qiaquick PCR purification kit (Qiagen) and subjected to Big Dye terminator sequencing at the Australian Genome Research Facility (AGRF). Contiguous sequences were assembled using Geneious Version 7.1.5 (Biomatters, Auckland, New Zealand) and compared with existing tick COI sequences in GenBank using the Basic Local Alignment Search Tool (BLAST).

### Detection of *T. orientalis* by quantitative PCR

Ticks grouped by species or phylogenetic cluster, and where possible by host and location, were dissected for quantitative PCR (qPCR) analysis by removal and collection of the capitulum and the anterior portion of the scutum. Separate sterile forceps and scalpel blades were used for the dissection of each tick. For mosquitoes and march flies, tissue from the head, abdomen and thorax was extracted. Tick tissue was pooled in groups of 1–10 samples depending on tissue volume to yield a total mass of 10–15 mg per pool. March flies (*Dasybasis* sp.) were tested individually by dissecting them laterally to yield 10–15 mg of tissue. Mosquitoes were also tested in 10–15 mg pools. Thirteen pools of mosquitoes were tested (approximately 10 mosquitoes per pool). All remaining mosquitoes were batch tested. DNA extraction was performed as described above for the tick legs, except that each tissue sample was homogenised prior to extraction with a sterile microfuge pestle. qPCR was performed using a validated multiplex qPCR assay for *T. orientalis* detection and genotype differentiation as described previously [36]. This assay was used for quantification of the total load of *T. orientalis* (Universal) and to semi-quantitatively determine the relative proportions of the Ikeda and Chitose genotypes (UIC assay). DNA extracts were also tested for the Buffeli genotype of *T. orientalis* using a semi-quantitative singleplex qPCR (B assay). For this assay, the same reagents and instrumentation described in Bogema et al. [36] were used, however, the probe mix was substituted for a single probe design based on an *in silico* analysis to specifically target the Buffeli genotype (5′ - FAM-CTCCTTTGCAGTATTCTTCTATCTC-BHQ1 - 3′). The analytical specificity of the Buffeli probe for this genotype was assessed using plasmids containing Ikeda Chitose or Buffeli MPSP gene inserts and also DNA extracts from *T. orientalis*-negative cattle and cattle infected with the closely related parasites, *Babesia bovis* and *B. bigemina* all as described in Bogema et al. [36]. The limit of detection (LOD) was defined as the limit where 95% of assays were successful and was experimentally determined by testing 8 replicates of Buffeli plasmid DNA at dilutions 15000, 1500, 150, 50, 15, 5, 1.5 and 0.5 gene copies/μL (GC/μL), followed by Probit analysis. The efficacy of the Buffeli singleplex assay was confirmed on bovine blood extracts previously demonstrated to be positive for this genotype [9]. A standard curve generated from a 10-fold dilution series of a plasmid containing the Buffeli MPSP gene [36] ranged from 1.5 × 10^1^ to 1.5 × 10^7^ to MPSP GC/μL and was included in each run. Quality acceptance parameters for standard curves were an R^2^ value > 0.98 and an amplification efficiency between 90 and 110%.

## Results

### Identification of tick species

**Figure 2:**
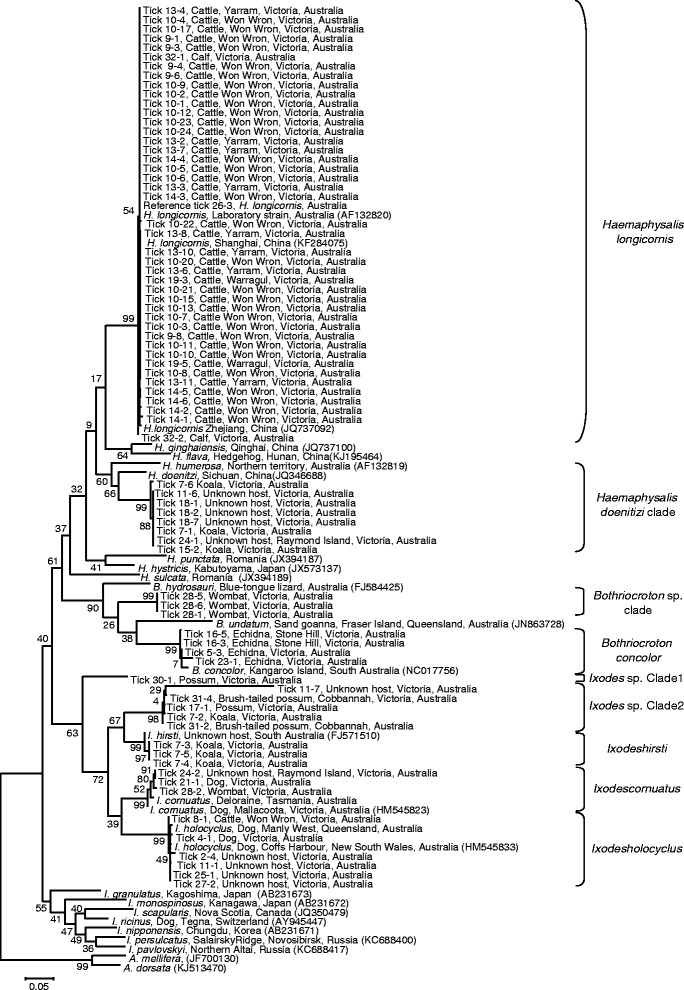
Phylogenetic tree of ticks investigated. Phylogenetic tree based on mitochondrial cytochrome oxidase I (COI) gene sequences of ticks collected in this study along with reference tick COI sequences sourced from Genbank. The phylogenetic tree is based on a MUSCLE alignment of the sequences in which gap positions were trimmed and was constructed using the Maximum Likelihood method based on the model of Tamura and Nei, within the program MEGA v6 [41]. The tree with the highest log likelihood is shown. Bootstrap replications (1000) were performed, which are expressed on each node as a percentage. The scale bar indicates the number of nucleotide substitutions per site. Accession numbers for references sequences are indicated. Sequences derived from this study were deposited in GenBank (Accession numbers - to be provided). Species (or clades) identified in this study are indicated on the right.

Morphological identification and molecular testing aligned for all specimens examined. A number of ticks were not examined morphologically due to damage to the tick during collection limiting our ability to make an accurate identification. A total of 81 tick COI barcodes were identified; 56 from ticks of the *Haemaphysalis* genus, 18 from *Ixodes* spp. and 7 from *Bothriocroton* spp. A phylogenetic tree constructed from a MUSCLE alignment of these sequences and GenBank reference sequences is shown in Figure 2. Tick barcode sequences clustered into 9 major clades, 8 with high (>97%) bootstrap support. Species identified included *H. longicornis*, *B. concolor*, *I. holocyclus*, *I. hirsti* and *I. cornuatus*. The remaining 4 clades identified also clustered within the genera *Haemaphysalis, Bothriocroton* and *Ixodes*, however these tick samples could not be confidently assigned to particular species. Furthermore, the four unidentified clades included damaged ticks unsuitable for morphological identification. There was moderate bootstrap support for a relationship between *Haemaphysalis* ticks collected from koalas (*Phascolarctos cinereus victor*) and the species *H. doenitzi* and *H. humerosa*. Ticks collected from the common wombat (*Vombatus ursinus*) were of the *Bothriocroton* genus but formed a distinct cluster from *B. concolor* (the echidna tick), and *B. undatum* and *B. hydrosauri*, which are typically found on reptiles. A further cluster of sequences forming a sister group to *I. hirsti* was identified from both koalas (*P. cinereus* victor) and brush-tailed possums (*Trichosurus vulpecula*)*.* Tick sample 30–1, also collected from *T. vulpecula*, was most closely related to members of the *Ixodes* genus, but did not cluster with any other species for which sequence data was available.

### *Theileria* qPCR

The analytical specificity and sensitivity of the *T. orientalis* multiplex UIC assay has been reported previously [36]. The analytical specificity of the B assay was confirmed using both plasmids containing MPSP genes and Buffeli positive and negative EDTA blood extracts (data not shown). The limit of detection of the Universal, Ikeda and Chitose components of the UIC assay are 17 (8–45), 27 (8–90) and 20 (6–65) GC/μL respectively [36]. The limit of detection of the B assay used in this study was found to be similar at 20 (9–43) GC/μL. Samples yielding amplification above these levels are reported as positive (Table 1). Amongst the ticks examined, only *H. longicornis* was found to be positive for theilerial DNA and these were collected from cattle in the districts of Won Wron, Yarram, Bairnsdale and Warragul (Figure 1), all areas in which clinical outbreaks of theileriosis have been recently reported [37,38]. In 2013, a serological and haematological survey found evidence of theilerial infection in 10 of 15 herds from these regions of Gippsland [36]. As shown in Table 1, ticks sourced from these regions were found to harbour all 3 genotypes of *T. orientalis* examined (Ikeda, Chitose and Buffeli). The Ikeda genotype, which has caused clinical outbreaks in Australia [6,9,38] was found in all pools of ticks testing positive for *T. orientalis*. The Buffeli and Chitose genotypes were found in all, and all but two, of the *T. orientalis*-positive pools respectively. These results are consistent with prior studies on blood samples collected from cattle in Eastern Victoria, in which all three of these genotypes were variably detected in disparate herds [37,38]. Quantitative data indicated that parasite load was highest in ticks sourced from Won Wron and Yarram and in most cases, the Ikeda genotype was present in higher concentrations than either the Chitose or Buffeli types (Table 1). Because pools of ticks were tested, this may equate to high levels of the Ikeda parasite in individual ticks or an increased prevalence of the Ikeda type within multiple ticks.

**Table 1:** **Tick species, host and location and results for** ***Theileria orientalis*** **universal and genotype specific qPCR**

**Tick species or clade**	**No. ticks/ pool**	**Host - Location**	**qPCR result**			
			**(GC/μL DNA extract)**			
			***Theileria orientalis***	**Ikeda**	**Chitose**	**Buffeli**
*Haemaphysalis longicornis*	5	Cattle - Won Wron	8.7 × 10^3^	+ (7.4 × 10^3^)	-	+ (2.7 × 10^3^)
*Haemaphysalis longicornis*	5	Cattle - Won Wron	0	-	-	-
*Haemaphysalis longicornis*	5	Cattle - Won Wron	0	-	-	-
*Haemaphysalis longicornis*	5	Cattle - Won Wron	1.5 × 10^4^	+ (7.1 × 10^3^)	+ (2.9 × 10^3^)	+ (4.5 × 10^3^)
*Haemaphysalis longicornis*	6	Cattle - Won Wron	4.2 × 10^3^	+ (1.5 × 10^3^)	+ (1.5 × 10^3^)	+ (1.1 × 10^3^)
*Haemaphysalis longicornis*	6	Cattle - Won Wron	1.0 × 10^4^	+ (4.6 × 10^3^)	+ (1.8 × 10^3^)	+ (3.3 × 10^3^)
*Haemaphysalis longicornis*	5	Cattle - Warragul, Bairnsdale	1.9 × 10^2^	+ (6.3 × 10^1^)	+ (1.0 × 10^2^)	+ (3.5 × 10^1^)
*Haemaphysalis longicornis*	5	Cattle - Yarram	4.1 × 10^4^	+ (3.6 × 10^4^)	-	+ (1.4 × 10^4^)
*Haemaphysalis longicornis*	4	Cattle - Yarram	9.2 × 10^2^	+ (4.2 × 10^2^)	+ (2.5 × 10^2^)	+ (2.5 × 10^2^)
*Haemaphysalis* sp. *(H. doenitzi* clade)	5	Koala - Raymond Island	0	-	-	-
*Haemaphysalis* sp*. (H. doenitz*i clade)	3	Koala - Raymond Island	0	-	-	-
*Ixodes holocyclus*	6	Cattle - Bairnsdale	0	-	-	-
		Dogs - Bairnsdale				
*Ixodes cornuatus*	10	Dog - Bairnsdale	0	-	-	-
*Ixodes cornuatus*	2	Wombat - Raymond Island	0	-	-	-
*Ixodes* sp. (clade 2)	5	Possum, Koala - Raymond Island, Bairnsdale	0	-	-	-
*Ixodes hirsti*	3	Koalas - Bairnsdale	0	-	-	-
*Ixodes* sp*.* (clade 1)	1	Possum - Fernbank	0	-	-	-
*Bothriocroton concolor*	5	Echidnas - Heyfield	0	-	-	-
*Bothriocroton concolor*	5	Echidnas - Heyfield	0	-	-	-
*Bothriocroton concolor*	2	Echidnas -Bairnsdale, Orbost	0	-	-	-
*Bothriocroton sp.* clade	3	Wombat- Bairnsdale	0	-	-	-

Several thousand mosquitoes were collected on outbreak farms or within close proximity. The mosquitoes identified include; *Aedes camptorhynchus, Aedes notoscriptus, Coquillettidia linealis, Culex australicus,* and *Culex molestus.* Of the 13 pools of mosquitoes tested, one was weakly positive for *T. orientalis* (41 GC/μL), with only the Buffeli genotype identified over the limit of detection of the assay. Batch testing of the remaining mosquitoes also resulted in a weak positive (20 GC/μL) for *T. orientalis* with no genotypes exceeding the limits of detection for the respective assays. Biting flies (*Dasybasis* sp.) tested (n = 28) were found to be negative for *T. orientalis* and all genotypes.

## Discussion

The epidemiology of the *T. orientalis* outbreaks in south-eastern Australia is largely unknown, although outbreaks in Victoria are believed to be linked to introduction of infected cattle from New South Wales to a farm near Seymour [7], at the periphery of the known range of *H. longicornis* (Figure 1). Prior evidence for the transmission of *T. orientalis* by *Haemaphysalis* ticks in Australia has been contradictory, but *H. longicornis* ticks sourced from both Japan and Australia have been shown experimentally to transmit the Japanese *T. orientalis* Ikeda genotype trans-stadially [2]. Indeed, the *H. longicornis* barcoding sequences obtained in this study suggest that the *H. longicornis* populations in Eastern Victoria are relatively homogenous and are closely related to those in Asia. Interestingly, the known range of *H. longicornis* in Australia closely mirrors areas in which outbreaks of clinical theileriosis have occurred, including an isolated population of this species in the south west of Western Australia (Figure 1). It could be hypothesised from the prior data that *H. longicornis* is a likely vector for *T. orientalis* Ikeda in Australia and the detection of *T. orientalis* Ikeda DNA in ticks from various regions in Victoria supports this view. Based on previous studies, the ability of *H. longicornis* to transmit *T. orientalis* Buffeli is less certain [22]. It is noteworthy therefore, that the Buffeli genotype (as well as the Chitose genotype) was detected within *H. longicornis* ticks along with the Ikeda genotype. This study also revealed *H. longicornis* as the major tick species found on cattle in Victorian herds suffering recent clinical outbreaks, further implicating this species as a likely vector of bovine theileriosis. The location of the detected theilerial DNA within *H. longicornis* is the focus of continuing research. Furthermore, extensive analysis of the proportions of the *T. orientalis* genotypes within individual ticks would be of future interest to determine whether particular genotypes are selected during the tick phase of the parasite’s lifecycle [39].

Other *Haemaphysalis* and *Ixodes* spp. have recently been implicated in transmission of *T. orientalis* in the Eastern Hokkaido and Okinawa prefectures via molecular screening of tick species for the presence of the parasite [11]. Interestingly, we did not detect theilerial DNA in any other tick species or march flies examined in this study. Two pools of mosquitoes were weakly positive for *T. orientalis*; however because these samples were close to the limit of detection of the assay, only the Buffeli genotype was identified in one of the pools. Further research is required to confirm their role as possible mechanical transmitters, as has been demonstrated for lice in Japan [40]. Studies are currently underway to determine the volume of blood required to transmit infection, and this will give an indication whether biting arthropods could pose any risk for mechanical transmission of the disease in Australia. The number of haematophagous flies caught on the outbreak farms was too small to be conclusive, and lice should also not be ruled out as a possible vector, even though no herd in the outbreak regions investigated showed evidence of louse infestations at the time of theilerial diagnosis. An understanding of the epidemiology, including the identification of vector(s), mechanical and intermediate hosts, is essential to stem the outbreaks of this emerging disease, and is the subject of ongoing investigation.

## Conclusions

This paper presents the first data identifying DNA from *T. orientalis* in *H. longicornis* from herds with recent outbreaks of clinical theileriosis. This adds to the body of international evidence for its role in the life cycle and transmission of the parasite. Further studies are in progress to demonstrate the sporozoite stage of the parasite within *H. longicornis* and to confirm transmission of the various *T. orientalis* genotypes from *H. longicornis* to cattle.
